# Wearable technologies in osteoarthritis: a qualitative study of clinicians’ preferences

**DOI:** 10.1136/bmjopen-2015-009544

**Published:** 2016-01-25

**Authors:** Enrica Papi, Ged M Murtagh, Alison H McGregor

**Affiliations:** 1 Department of Surgery and Cancer, Imperial College London, Charing Cross Hospital, London, UK; 2 Department of Surgery and Cancer, Imperial College London, St Mary's Campus, London, UK

**Keywords:** Rehabilitation, wearable technology, Osteoarthritis, clinicians, interviews

## Abstract

**Objective:**

This study investigates clinicians’ views of health-related wearable technologies in the context of supporting osteoarthritis (OA) long-term management. Clinicians’ preferences are critical in identifying realistic implementation strategies for such technologies.

**Design:**

Qualitative study incorporating an inductive thematic analysis applied to identify key themes from clinicians’ responses.

**Participants:**

Clinicians, including 4 general practitioners, 4 physiotherapists and 5 orthopaedic surgeons were interviewed.

**Setting:**

The study was conducted in a University setting.

**Results:**

Participants all agreed wearable technologies could positively complement their role and enhance their relationship with patients. Perceived benefits of wearable technologies included monitoring patients’ progress, treatment evaluation, monitoring compliance and informing clinical decision-making. The device should be designed to provide objective data of patients’ locomotion capability in an easy and timely fashion via a simple interface. Data should be available to both clinicians and patients to provide them with the motivation to achieve clinical goals and allow them to take ownership of their treatment. The use of technology was also seen as a way to more effectively plan treatment and manage patients’ contact time saving time and cost.

**Conclusions:**

Findings support the use of wearable technologies to enhance current OA management and suggest clinical uses. Adoption of technologies could have implications on the effectiveness of treatment provided overcoming current barriers, in particular compliance with treatment.

**Strengths and limitations of this study**

- This is the first study to evaluate the use of wearable technologies from a clinical perspective in the context of osteoarthritis care.
- In-depth interviews allowed an understanding of clinicians’ preferences to identify adoption strategies of wearable technologies within an osteoarthritis rehabilitation framework.
- Osteoarthritis requires different categories of health professionals involved in its management and these were interviewed in this study.
- Results are based on a small sample size of clinicians recruited within the London National Health Service (NHS) area.

## Introduction

Osteoarthritis (OA), among other musculoskeletal conditions, is one of the most common causes of long-term adult disability.^1^ Eighty per cent of those affected report some degree of functional limitation and 25% cannot perform major daily living activities.^2^ The prevalence of OA is set to rise along with its economic burden both from direct and indirect associated costs, which are already high.^3^ ^4^

A paradigm shift in the management of long-term conditions, such as OA, towards self-management strategies has been advocated to reduce the patient and societal burden of such diseases.^4^ ^5^ For OA, guidelines recommend rehabilitation based on exercise programmes to mitigate symptoms and disease progression. However, the effectiveness of these protocols is highly dependent on supervision and compliance, which is often poor.^6^ Recent advancements in health-related measuring technologies could offer new opportunities for delivery of rehabilitation programmes outside a clinical setting, allowing remote monitoring and feedback of key measures to both health professionals and patients.^7^ ^8^ These portable devices could potentially enable patients to become more active in the management of their condition and fulfil their interest in personalised health information while also aligning with the current focus on patient self-management^7^ as well as the need for more accurate objective measures of patients’ functions.^9^

These devices are designed to record quantitative data in a mobile environment, embedded in the user’s clothing or fitted as an accessory.^10^ Numerous devices have been developed to date aimed at monitoring patient ambulatory performance for rehabilitation purposes, but their uptake and acceptance in clinical environments remains poor.^11–13^ The uptake of these technologies is influenced by their intended use, perceived usefulness, ease of learning, success in early experimentation, right fit to a specific clinical context as well as user needs.^13–15^ Therein lies a problem. The development of these technologies has been largely driven by engineering requirements.^12^ Consequently, less attention has been devoted to users’ preferences.^11^ ^12^ Recent research conducted to address this gap in knowledge has focused on examining patients’ preferences for wearable technologies. By contrast, comparatively scarce attention has been given to health professionals’ views of these devices.^12^ ^16^ Therefore, we know relatively little about how these devices might work in the context of clinical practice. Health professionals, like patients represent a key user group. Unlike patients, however, they possess knowledge and insight of clinical practice, which would be critical in identifying realistic implementation strategies. Moreover, health professionals could assist with promoting acceptability among patient groups.

Since each device should address the complexity of a targeted condition, our initial focus has been the use of wearable systems in the context of guiding and supporting OA rehabilitation. OA requires different categories of health professionals involved in its management.^4^ We asked general practitioners (GPs), physiotherapists and orthopaedic surgeons their views of wearable technology and how they would use it in their practice. The aim of this study was to investigate health professionals’ perspective of wearable technology and draw on the findings to develop a wearable system for OA rehabilitation. The findings from in-depth interviews are reported.

## Methods

### Study design

This study was a qualitative study based on in-depth interviews with health professionals. All participants provided written informed consent form prior to each interview.

### Participants

A total of 13 health professionals (age range 35–74 years) were recruited for the study within the London National Health Service (NHS) area. Health professionals were invited to take part by email invitation sent over a year period starting from January 2014. The participants consisted of four physiotherapists, four GPs and five orthopaedic surgeons. All participants had at least 7 years of experience in their specialty up to 40 years of practice. Physiotherapists were specialised in musculoskeletal conditions, four out of five surgeons were specialised in hip and knee joint surgeries and one in spine surgeries. GPs practised in different areas of London covering more to less affluent neighbourhoods.

### Interviews

Semistructured interviews were conducted by two researchers (EP and GMM). Interviews were tape recorded and transcribed verbatim. Interviews lasted between 30 and 45 min. Participants were asked a series of open-ended questions formulated to explore their knowledge and views of wearable technologies (box 1). Interview questions were developed to touch issues of technologies uses, impact on practice and fit on clinical routine with particular reference to practical issues of what to measure and feedback. A brief introduction on the scope of the interview and research project was provided at the beginning of the interview session.

Box 1 **Interview open-ended questions**

- Contextual background
  1. What is/can you describe your professional role?
  2. What types of patients do you come into contact with?
    - Is there a particular type/category?
    - Does it vary?
    - How many patients do you see per day?
    - Is there a particular age range of patients you work with?
  3. Does most of your work involve assessing patients’ mobility?
  4. What is the general remit of your work?
- Wearable technologies
  1. Do you know anything about wearable devices?
  2. If so tell me what you know
  3. How are they used?
  4. From what you know, what's your view of them?
  5. How would such a device help you in your own work?
  6. How would you use them? Or if you can think of one use which will be the most appropriate for you?
    - Do you think such a device would be good for patients? Would you use with all patients?
    - If this device could offer some functionality that you think may be important what would that be?
    - Is there anything you would like to monitor in particular?
  7. How would you ideally record the data and how would you like them to be available to you?
- Impact of wearable technologies on current practice
  - Can you envisage a way that such a device might negatively impact on your work?
  - Do you think wearable devices could replace part of the routine process patients are treated with?
  - From your professional perspective can you describe what you would want from such a device?
- Closure
  - Is there anything else that you would like to add about wearable devices that we haven't covered here?

### Qualitative data analysis

The transcribed data from the interviews were analysed using an inductive thematic analysis process^17^ to ensure, where possible, that the analysis was data driven and not wholly driven by theoretical or analytic interest. First, EP and GMM examined the interview data to establish preliminary themes, which were extracted through careful examination of the respondents’ accounts. Second, these preliminary themes were reanalysed by EP and GMM to ensure a closer alignment with participants’ responses and to establish main recurrent themes. Finally, EP and GMM examined each other's data set to facilitate consistent interpretative analysis of the data and clarify the key findings.

## Results

All three professional groups highlighted the potential of using wearable technologies in OA management to improve service and patients’ experience. All were able to provide examples of wearable technologies either coming from personal use or general knowledge. Overall wearable technology was seen as a tool to support and solidify objective measures of treatment planning to guide and motivate patients. The analytical process revealed four main recurrent themes: utility, doctor–patient relationship, design specification and critical aspects/negative impact on practice. These themes are described below and verbatim comments are indicated using the following notation: P to indicate physiotherapists, OS to indicate orthopaedic surgeons and GP denotes general practitioners.

### Utility

Interviewees extensively commented on how the technology could be used in clinical practice. The main uses are summarised in box 2.

Box 2 **Utility of technology in clinical practice**

- Progress monitor
- Realistic assessment/support treatment planning
- Motivate patients
- Efficient management
- Treatment evaluation
- Monitor compliance

All participants agreed that the technology would not play a major role in diagnostic assessment and rather than replacing a clinician's role it would support it. Clinicians considered the devices as tools that could provide objective measures of clinical changes as adjunct to the subjective account from the patient of their level of pain, movement and activity.

Surgeons have been diagnosing for a long time. The device is not really going to help with diagnosis. (OS2)

Add on, support to what we do, I do not think you can negate what physios do especially with OA. It will give nice objective sense of where the patient is at..but you cannot move away from actually having someone listening to what you are saying. (P4)

Traditional assessment tools would remain as the mainstay for diagnosis especially for physiotherapists and surgeons; however, GPs suggested that they could use the data for referral decisions:

If we see someone with a knee problem we're not going to refer all of them on to an orthopaedic team. As a tool for discriminating which patients should be referred or not it could be useful. (GP3)

#### The provision of patient data and clinical decision-making

The provision of objective data by these devices was recognised as a tool to help, devise treatment plans and support clinical decision-making. Both the physiotherapists and surgeons held the view that a wearable device could be a measure of clinical treatment that would be objective and evidence based to support analytical practice:

Objective records so that treatment could be planned based on that and then see what happen after you change the gait. (OS5)

It[the device] would enable us to become more analytical…Having something where we can actually say look we've made a change with this intervention and here's the data that would be nice…we're going to need more scientific backup for the things we do. (P4)

The device would be able to provide an objective measure of progress and mobility in contrast to the subjective account of the patient that could be now checked against the objective data provided by the device:

Objective data rather than subjective as the one picked up in the clinics and of what the patient is telling you; whereas the device will bring you more real time objective data analysis and hopefully this will give you a clear understanding and idea of how the patient is doing. (OS3)

You have to start with their subjective data and see if that is supported by more objective data. (GP1)

Data will allow clinicians to objectively measure improvement which has been poorly defined to date:

For us at the minute objectify what better means is quite difficult whereas having something more scientific to analyse would make mapping their progress a little more clinical significant for them. (P4)

Moreover, it will allow an assessment not confined to a visit snapshot:

If someone is watching you walking you do not walk with a normal gait, do you?…it will be an adjunction sort to confirm what your suspicious were, so as much as you send someone for MRI you could look at that data and say well actually in the clinic he was limping but actually when is out and about his walking is not too bad…it giving you that sort of fly in the wall sort of perspective of the patient so that you could see what’s happening when they are not thinking about you being there. (GP2)

In supporting clinical decision, some respondents also described the technology as a tool that could evaluate the effectiveness of the treatment provided. In this context, it could be the success of a surgical intervention or exercise programme:

…hardcore evidence of how different operations affect patients. (OS1)

The core functionality was identified in patients monitoring. The benefit from the monitoring is twofold: (1) to motivate patients; (2) to improve OA management effectiveness. In relation to the former one physiotherapist commented:

Progress tracker will hugely improve their motivation and would be really nice to have this record. (P1)

#### Managing referral and patient contact time

Improving management effectiveness was a particular focus for GPs and physiotherapists where patients’ visits are numerous, frequent and affected by time constraints. Technology could assist in making the OA care process more efficient. The additional information that it brings along *can be used to book appointment more wisely when necessary, maximising clinicians and patients time*:

Waiting list are high whereas with something like that I could say to them look you ring in and tell me what you are recording…it may reduce the amount of contact time we need with patients and they are satisfied because they are achieving what they need to. (P3)

It followed to some extent that the technology was considered as a means to accelerate recovery:

If a patient using the device have done all your exercises…They may then contact and say your device you gave me told me I am actually doing really well, can I now progress? Rather than waiting too long and not progressing soon enough. (P4)

#### Compliance

Poor patient compliance to treatment course emerged from the interviews. As already mentioned, technology could motivate patients and along with day-by-day ‘surveillance’ could impact positively on compliance to treatment.

This device can allow monitoring, once you monitor them you can tell they are not doing their exercise and gear them up a bit, patients have other commitments, other pressures so sometimes their rehabilitation doesn’t happen so that’s a problem we need to sort out, compliance is the biggest problem. (OS3)

Patients have awful compliance with exercise diaries having something where you can't fiddle and I would be able to see what you've done and how many times you've done it and how well… (P4)

### Doctor–patient relationship

On the whole, all the professional groups were of the view that wearable technologies monitoring the patients would enhance rather than interfere with the doctor–patient relationship:

The device would objectify the patient/doctor relationship but in a positive way. The patient would feel you know more about them…they would then know that I care more because I know that bit more about them…and your chances of making a mistake are reduced. (OS1)

The enhancement of this relationship can reassure patients that they are receiving high-quality treatment:

A lot of this people are generally told you've got arthritis it is a fact of life, get on with it, and so they kind of feel a bit lost as if none actually watching them and monitoring them and giving them any help and assistance in what they should or should not be doing and so something like that may offer them almost that someone is listening to me someone cares about how I am getting on and how I am doing on a daily basis, how my condition is progressing.. (P4)

#### Ownership of information

Ownership of the information from the device has to be with the patient. The patient should be able to take ownership of the information and use it to their advantage. (GP1)

Having more information to hand will give patients’ the opportunity to be more in charge of their condition promoting self-management. However, supervision will always be ensured:

I have control and the patient has control, what you do not want to happen is the patient to go away with the device and overdoing something or underdoing but overdoing create more pain. (P3)

With the use of technology both health professionals and patients will be equally involved in their OA treatment. This will allow improved organisation of the management and provision of care; planning appointments only when necessary with the backup of objective information in home environment:

It would allow remote assessment and so save patients having to travel into hospital…it would be a bit like bringing the lab out to the patient's home. (OS3)

### Design specification

In terms of appearance, all participants agreed that the technology should be wearable, small, easy to get on and off, robust, discrete and aesthetically preferable:

People do not like to know they are on show. (P2)

It should not inhibit movement and be “easy to remember to carry around” (P1). Moreover it should be simple to operate, user-friendly and “viable for both young and older group” (P2) while still maintaining a level of adaptability to different people to use. Feedback should be available to both patients and clinicians and it was suggested to use an app for tablets/smartphones. The app interface should be simple with mainly graphical visualisation of data processed, “not much language” (GP1), “no raw data” (OS5), “brief summary report” (GP3) and allowing “little interaction with the user as possible” (P2). Patients’ reports should be received by clinicians automatically “at different time frames according to the stage and use of the patients” (GP4) but with “alarms flagging up problems” (OS4).

From the interviews, it emerged how technology should be specific for the condition targeted. This dictates positioning,

A wearable device if it is for orthopaedic it is all about movement so it has to be close to the joint, placed in the lower limb. (OS3)

as well as what to measure:

Blood pressure, pulse not useful for what we are doing here as musculoskeletal physios. (P1)

With regard to what to measure GPs emphasised they would like “simple measures of improvement or deterioration” whereas physiotherapists and surgeons would like more specific details and quantification of patients’ locomotion. These included distance, step length, speed, cadence, symmetry between legs, muscle activation, joint loading, ground reaction force, range of motion at selected joints, joint angles:

At the moment we can only infer patients metrics (e.g. knee angles). If you don’t have enough knee flexion you cannot run that fast. OA knee is less flexible we need to know how much they are doing. (OS1)

An overall interest was also expressed in general activity level, activities performed, ‘time spent on their feet as opposed to seating’, ‘How much they can do? How much do they do?’, ‘number of time they perform an exercises instructed’. It was also pointed out the importance of being easily quantifiable information ‘where clinicians can tap in targets’.

### Critical aspects/negative impact on practice

The most prevailing limitation to adoption was identified in the time required for managing the technology. This would include time to interpret and understand the data:

You need time to get used to it and interpreting what you need to look for. (OS3)

And time to instruct patients on how to use the technology:

It should be other people to handle the equipment and use it and know how to help the patients with it because our workload is already busy. (GP4)

Cost-effectiveness was also critical for adoption:

I am not sure how far the technology is today and also if the cost is at a level that is affordable for the NHS. (GP4)

However, this could be overcome:

Cost may be an issue but not insurmountable, if it is worth it you can justify it. (OS5)

Patients’ acceptance will play a role in the continuous use of the device:

Compliance is a real issue as I wonder if patients will actually wear these devices at home. (OS3)

To this, it adds on a counter motivational effect the device could have on patient:

It might show up what they're not doing rather than what they are doing and for some that could be a de-motivator, so choose the right people to use it with. (P2)

Success will hence be achieved by targeting the patients to who the device could be given:

…Select patients to give it to, I do not want to waste their time and my time, it is the same of any intervention, there is a bit of judgment call and deciding who you are giving it to. (P4)

## Discussion

Understanding the actual context of use of wearable technology against the background of patients’ knowledge and awareness of their treatment and progress, is critical to gathering a deeper understanding of the willingness to use technology in everyday clinical practice. Relatively few studies have examined health professionals’ acceptance of health-related wearable devices and, to our knowledge, none have reported on their use within the context of OA management from a clinical user perspectives.^12^ ^14^ ^18–21^ Despite studies reporting on the use of technology for the OA population, the majority of these studies were conducted in research settings in preclinical phases and did not report users’ preferences and how to use the information obtained in routine clinical practice.^11^ ^22–24^ The findings from our study indicate how technology could prove beneficial for health professionals groups touching on issues of patient management, doctor–patient interaction, compliance, feedback and impact on practice. By investigating health professionals’ viewpoints, this study provides novel insights on how technology could be integrated into current OA care along with design recommendations to be met by developers.

All three groups, physiotherapists, surgeons and GPs, were in agreement that technology would not replace their role but will offer support and enhancement to patient care. This was also the case for conditions other than OA.^18^ ^19^ Participants agreed that technology could add a limited value to the diagnostic process but its value could be proved in long-term management, offering monitoring and objective measures of patients’ status, if implemented appropriately. Adoption of technology in current practice was related, in the first instance, to its ease of use and its effectiveness in providing easy to interpret and timely data to clinicians. These factors were seen as critical in encouraging patient compliance to use the technology. However, health professionals can play a critical role in enabling implementation. For example, participants reported that non-compliance with the technology could be avoided by careful selection of patients who are more likely to positively receive the technology and make the most of its use. In another study conducted by some members of this research team, it was found that patients with OA from different age and sociodemographic groups were enthused by the technology and highly motivated to use it to support the management of their condition.^16^ Aligning these preferences with the clinicians’ preferences outlined in this study would appear to be a solid prospect for developing adoption strategies within clinical settings.

Our study highlights clinicians’ views on how these devices could help improve compliance to treatment and self-motivation, issues well documented in the literature.^25–27^ In addition, the provision of objective data could enable goal setting and progress monitoring both critical components of any long-term treatment programme.^27–30^ It is important, however, that measures of performance/goals are tailored and personally meaningful to the patients and their conditions.^14^ ^21^ ^28–30^ Participants suggested variables they would like to monitor in relation to OA to be able to use such technologies to map patients’ progress and inform treatment. GPs suggested they would prefer simple measures of improvements whereas both physiotherapists and surgeons would prefer more detailed information of locomotion capability. Participants recommended that the measurable outputs from these technologies should be easily quantifiable to establish specific goals relating to locomotion performance (eg, joint loading, step length, range of motion, sagittal angles) and not just to activity levels. Advocated technologies should be able to measure and monitor such variables and report on them periodically in the form of graphical reports available to both health professionals and patients.

Remote monitoring and patient self-monitoring was conveyed as an approach that could allow better time management and reduce unnecessary hospital appointments. A hint of more effective care provision and recovery process emerged from the interviews. Hospital visits could be planned when necessary with the back-up of technology data, flagging positive and critical situations. A more evidence-based practice could be developed with the technology, which allows clinicians to evaluate treatment options, and thus maximises effectiveness of the interventions. The likelihood of reducing appointments and enhancing patient function, through an inexpensive technological approach, could save time and cost. Joint replacements, days at work lost, medication and comorbidities could all be reduced if care is effectively delivered saving money to the healthcare systems and patients themselves.^4^

The continuous exchange of information between patients and clinicians was envisaged to reinforce the patient/doctor relationship while providing patients with the opportunity for more independent management of their own condition. This is in line with patients’ views on how technology and more information could allow them to take control and seek clinical support when necessary, pacing care to their needs and convenience.^16^ The technology could provide that sense of supervision and continuous guidance during treatment which is otherwise lacking in current practice.^16^ A recent paper highlighted the gap in OA management at early stages until surgery remains the only option.^31^ The authors call for new solutions to be implemented that need to be personalised and actively involve both patients and clinicians. Current guidelines reflect this need promoting exercise and self-management to improve patients’ function.^5^ Technology as a tool for self-management could offer this opportunity if both patients and clinicians’ needs are targeted and cooperate harmoniously.

We acknowledge the limitations of these findings, that they are based on a small-scale interviewees group from the London NHS area. However, the group comprised differing healthcare practitioners, all involved in the care pathway of patients with OA, to allow a more general viewpoint. Their views were similar and where major distinctions were noted they have been highlighted. A good level of data saturation was achieved, but in light of the small group, a broader generalisation of the findings may require more participants from other areas than London. Nevertheless, this paper opens up unspoken issues that could help in the development of new technologies-based approach for OA rehabilitation meeting a clear clinical need in this field and foraging for new collaborations between technology engineers and end-users.

In conclusion, the perceived usefulness and uptake of wearable technology is critically influenced by its specificity to the targeted condition.^13–15^ However, effective implementation of any technology in a given context should feature the views of potential users. We have investigated views on usability and acceptability of health-related wearable devices with a small group of health professionals in the context of OA. Overall, the findings support the use of technology to assist treatment of patients with OA to enhance current care.
